# Supplementary material for: Second Victims among Austrian Pediatricians (SeViD-A1 Study)
Source: Healthcare (Basel). 2023 Sep 8;11(18):2501. doi: 10.3390/healthcare11182501 (PMC10531173; doi:10.3390/healthcare11182501)
Supplement: Supplementary file 1 [file healthcare-11-02501-s001.zip › healthcare-2570244-supplementary.pdf]

Table S1. Factors contributing to the variance explanation of symptom load. Results of multiple linear regression, n=288.

Independent Variable	Unstandardized Regression Coefficient B	Srtandardized Regression Coefficient β	*p*	BCa 95%CI Lower	BCA 95% CI Upper
Constant	2.52		0.23	-1.59	6.44
Gender (female = 1, male = 2)	0.10	0.02	0.80	-0.74	0.91
Age[1]	0.04	0.19	0.37	-0.06	0.14
Professional experience (years)[1]	-0.07	-0.24	0.10	-0.16	0.02
Workplace (inpatient = 1, outpatient = 0)	-1.35	-0.13	0.01	-2.20	-0.39
Openness	0.41	0.11	0.01	0.09	0.76
Conscientiousness	0.06	0.02	0.80	-0.46	0.58
Extraversion	-0.39	-0.12	0.04	-0.78	-0.01
Agreeableness	0.23	0.06	0.33	-0.22	0.67
Neuroticism	0.78	0.22	0.0001	0.40	1.17

Outcome variable is symptom load caused by SV experience. [1] Predictor variable is mean-centred, Lower BCa 95%CI and Upper BCa 95%CI: lower and upper limits of 95% bias-corrected and accelerated bootstrapped confidence interval of unstandardized regression coefficient B based on 1,000 bootstrap samples, symptom load was computed using the participants' answers "Not at all"=0, "weakly pronounced"= 0.5, and "strongly pronounced"=1, the response option "I don't know" was considered a missing value of all 18 symptoms presented to the participants.
